# Longitudinal Associations between Self-Rated Health and Performance-Based Physical Function in a Population-Based Cohort of Older Adults

**DOI:** 10.1371/journal.pone.0111761

**Published:** 2014-11-03

**Authors:** Willa D. Brenowitz, Rebecca A. Hubbard, Paul K. Crane, Shelly L. Gray, Oleg Zaslavsky, Eric B. Larson

**Affiliations:** 1 Department of Epidemiology, University of Washington School of Public Health, Seattle, Washington, United States of America; 2 Group Health Research Institute, Seattle, Washington, United States of America; 3 Department of Biostatistics, University of Washington School of Public Health, Seattle, Washington, United States of America; 4 Department of Medicine, Division of General Internal Medicine, University of Washington, Seattle, Washington, United States of America; 5 School of Pharmacy, University of Washington, Seattle, Washington, United States of America; 6 University of Haifa, Mount Carmel, Haifa, Israel; University of Louisville, United States of America

## Abstract

**Background:**

Although self-rated health (SRH) and performance-based physical function (PPF) are both strong predictors of mortality, little research has investigated the relationships between them. The objective of this study was to evaluate longitudinal, bi-directional associations between SRH and PPF.

**Methods:**

We evaluated longitudinal associations between SRH and PPF in 3,610 adults aged 65–89 followed for an average of 4.8 (standard deviation [SD]: 4.4) years between 1994 and July 2011 in the Adult Changes in Thought study, a population-based cohort in the Seattle area. SRH was assessed with a single-item question in the ACT study. Participants were asked at each evaluation to rate their health as “excellent”, “very good”, “good”, “fair”, or “poor” in response to the question “In general, how would you rate your health at this time”. PPF scores (ranging from 0–16, with higher indicating better performance) included walking speed, chair rises, grip strength, and balance.

**Results:**

At the baseline visit, participants averaged 74.5 (SD: 5.8) years of age and 2,115 (58.6%) were female. In multivariable linear mixed models, PPF declined with age, with more rapid decreases associated with very good, good, and fair (vs. excellent) baseline SRH. Adjusted annual change in PPF was −0.17 points (95% confidence interval [CI]: −0.19, −0.15) for individuals with excellent baseline SRH and −0.21 points (95% CI: −0.22, −0.19) for participants with fair SRH. In multivariable generalized linear mixed models, lower baseline PPF quartiles were associated with lower odds of excellent/very good/good SRH at age 75, however, differences between baseline PPF quartiles diminished with age.

**Conclusions:**

These results suggest that less than excellent SRH predicts decline in physical functioning, however, poor physical functioning may not predict change in SRH in a reciprocal fashion. SRH provides a simple assessment tool for identifying individuals at increased risk for decline in physical function.

## Introduction

Self-rated health (SRH) is a widely used measure of general health status that is typically assessed by asking an individual to rate their health [1]. Poor SRH is a consistent predictor of mortality [2]–[7] and morbidity [8]–[11] even after accounting for objective health indicators. The mechanisms underlying these associations are poorly understood. However, one possible mechanism is through decline in physical function [12], [13].

**Figure 1:**
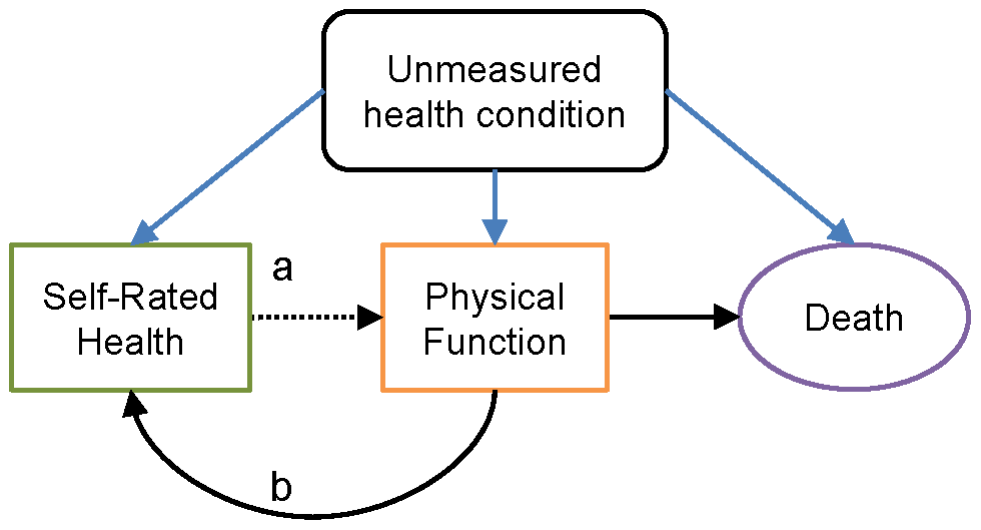
Hypothesized relationships between SRH and physical function. Poor SRH is likely a proxy for an individual's underlying health state that is not captured by other measures. SRH then is statically but perhaps not causally associated with decline in physical function and an increased risk of death (pathway a). Alternatively, poor physical function may lead people to rate their health as poor (pathway b).

Poor SRH is associated with faster decline in functional ability in older adults [8], [10]. In turn, impairment in physical function is considered an early stage in the disablement process [14] and is linked to mortality [6], [15], [16]. Thus, it is hypothesized that decline in physical function may be an intermediary between SRH and mortality [12], [13]. In this context, SRH may represent an individual's superior knowledge of their own health status and past health risks over objective health measures [1]. SRH is then perhaps statistically but not causally associated with decline in physical function and subsequently an increased risk of death [1] (Figure 1). However, SRH is likely a dynamic evaluation of health [4]. Individuals may rate their health as poor due to their current health status or recent changes in health [17]. Thus, poor SRH may be both a predictor and an effect of worsening health (Figure 1). Research on the relationship between SRH and physical function may clarify the mechanisms by which each acts as a predictor of mortality.

Few studies have examined longitudinal relationships between SRH and physical function in older adults. Ratings of fair/poor SRH have been associated with future decline in gait speed [12], [13]. Associations of SRH with decline in other measures of physical function have not been investigated. Summary measures of physical function, for instance, may be more robust than single items [18]. Furthermore, it remains unclear whether physical function predicts longitudinal change in SRH. Functional status has previously been associated with SRH but associations were found to be weaker for older adults [19]–[23]. Although SRH has been found to decline with worsening health [4], [24], other studies have found that SRH improves with age [25], [26]. Perceptions of health likely change with age [1]. Evaluating bi-directional associations between SRH and overall physical function may help elucidate factors influencing health perceptions and identify targets for early intervention.

The goal of this study was to evaluate bi-directional longitudinal relationships between SRH and performance-based physical function (PPF), a composite measure of tests that assess both upper and lower extremity function, among older adults [27]. PPF may capture functional limitations before difficulties are reported [28], [29]. In particular, we sought to quantify relationships between PPF and the subjective component of SRH beyond its correlation with health status. We hypothesized that poorer baseline SRH would be associated with more rapid decline in PPF (Figure 1, pathway a), and that poorer baseline PPF would be associated with more rapid decline in SRH (Figure 1, pathway b), after adjusting for other health indicators.

## Methods

### Study Population

Participants were enrolled in the Adult Changes in Thought (ACT) study, a population based prospective cohort study of incident dementia and Alzheimer's disease, previously described [30]. Briefly, participants were cognitively intact older adults randomly sampled from Group Health Cooperative members aged 65 and older in the Seattle area. The original cohort of 2,581 participants was enrolled between 1994 and 1996. Between 2000 and 2002 an additional 811 participants were enrolled, and in 2004 continuous enrollment began to replace participants who dropped out, developed dementia, or died. Individuals with dementia at baseline were not enrolled in the ACT study. All participants were followed biennially until time of dementia diagnosis, death, or drop-out. At baseline and biennial follow-up evaluations, data collected included demographic characteristics, medical history, cognitive function, memory functioning, blood pressure, depression, and physical functioning. The research protocol for this study followed the Helsinki declaration and was reviewed and approved by the Group Health and University of Washington institutional review boards. Written informed consent was obtained from all participants.

The current analysis included ACT participants aged 65–89 years old who were followed between 1994 and July 2011. The sample was restricted to participants with complete data on SRH, PPF, and covariates at one or more visits. For this study, we defined the baseline visit as the first visit with non-missing SRH, PPF, and covariate information. Follow-up of participants ended at time of dementia diagnosis, death or drop-out; and did not include the visit at which a participant was diagnosed with dementia due to concern that dementia may influence SRH and PPF measures.

### Measures

#### Self-Rated Health

SRH was assessed with a single-item question in the ACT study. This item is similar to SRH questions used in other questionnaires, particularly in the U.S. [1], such as the first question in the SF-36 health survey [31]. Participants were asked at each evaluation “In general, how would you rate your health at this time” with response options of excellent, very good, good, fair, and poor. The SRH question was asked before the physical function tests were performed. Although prior studies have used a variety of different wording and response options for SRH, they are considered to represent the same underlying variable [1], and concordance between different response options is good [32]. Prior studies have found dose-response relationships between original levels of SRH and adverse health outcomes, indicating the validity of SRH [7], [9]. In analyses when SRH was the primary exposure, we used SRH at baseline retaining the original five categories. In analyses where SRH was the outcome, responses were dichotomized into excellent, very good, or good (“healthy”) SRH vs. fair or poor (“unhealthy”) [33], [34].

#### Performance-Based Physical Function

The PPF score was created following methods developed by Wang and colleagues (2002) [28] for the ACT study population. The PPF score consisted of four performance measures that evaluate upper and lower extremity function: 10-foot timed walks (walking speed), five repeated chair stand time (chair rises), standing balance, and grip strength (in kilograms). These tests were chosen based on previously published research [35]–[37] and study logistics. Walking speed was tested by asking participants to walk a 10-foot distance at their usual speed, using assistive devices if needed. The average of two walks was recorded. Ability to rise from a chair was assessed by instructing participants to stand up from a straight-backed chair with their arms across their chest. Participants successful with one chair rise were then asked to stand up and sit down five times, as fast as possible. They were timed from the first sitting position to the final standing position. To test standing balance, participants were asked to stand close to a wall and were timed for how long they could stand with their feet side-by-side before touching the wall for support. Participants who were able to stand with their feet in the side-by-side position for 10 seconds were next asked to stand with their feet in a semi-tandem position for 10 seconds. Those able to maintain the semi-tandem position were then asked to stand with their feet in a full tandem position for 10 seconds. Grip strength was evaluated using a handheld dynamometer [JAMAR hydraulic hand dynamometer] and measured to the nearest 0.1 kg. Participants were asked to grip the handle as hard as possible using their dominant hand. The average of three attempts was recorded.

A score of 0 to 4 was determined for walking speed, chair rises, standing balance, and grip strength tests. For all tests a score of 0 was given if the participant could not complete the test. Scores of 1–4 for walking speed, chair rises, and grip strength tests were based on previously published sex-specific cutoffs, which corresponded to sex-specific quartiles in the ACT population [28]. Scores of 1–4 for standing balance were categorized based on ability to maintain standing in each position for at least 10 seconds. Score cutoffs are summarized in Table 1.

**Table 1:** Sex-specific Cutoffs for Scores (0 to 4) for Walking Speed, Chair Rises, Standing Balance, and Grip Strength Tests Based on Previously Published Scoring.

	Cutoff for Men	Cutoff for Women
Walking Speed		
0	Unable to complete	Unable to complete
1	>4.5 seconds	>5.0 seconds
2	4.5 - 4.0 seconds	5.0 - 4.0 seconds
3	4.0 - 3.0 seconds	4.0 - 3.0 seconds
4	≤3.0 seconds	≤3.0 seconds
Chair Rises		
0	Unable to complete	Unable to complete
1	>20 seconds	>21 seconds
2	17–20 seconds	18–21 seconds
3	11–17 seconds	12–18 seconds
4	≤11 seconds	≤12 seconds
Standing Balance		
0	Unable to balance side-by side	Unable to balance side-by side
1	Able to balance side-by-side, unable to balance semi-tandem	Able to balance side-by-side, unable to balance semi-tandem
2	Able to balance side-by-side and semi-tandem, unable to balance full tandem	Able to balance side-by-side and semi-tandem, unable to balance full tandem
3	Able to balance side-by-side, semi-tandem, and full tandem for 1–9 seconds	Able to balance side-by-side, semi-tandem, and full tandem for 1–9 seconds
4	Able to balance side-by-side, semi-tandem, and full tandem for 10 seconds	Able to balance side-by-side, semi-tandem, and full tandem for 10 seconds
Grip Strength		
0	Unable to complete	Unable to complete
1	<25.0 kg	<15.0 kg
2	25.0–30.0 kg	15.0–20.0 kg
3	30.0–40.0 kg	20.0–25.0 kg
4	≥40.0 kg	≥25.0 kg

The sum of the four test scores determined an individual's PPF score (range: 0–16); higher scores corresponded to better performance. In analyses with PPF as the outcome, we used the total PPF score at each visit as a continuous variable. In analyses where PPF was the primary exposure, we categorized the baseline PPF score based on the study sample quartiles. We used baseline PPF quartiles because the relationship between baseline PPF and change in SRH may not be linear. In exploratory analyses, individual PPF components were dichotomized based on study sample quartiles into scores of 3–4 (“better” function), which generally corresponded to 75% of the participants vs. scores of 0–2; except balance, which had a highly skewed distribution, and “better” function was limited to a score of 4.

#### Covariates

At study baseline and follow-up visits, information was collected on demographics, health status, and chronic health conditions. Demographic factors measured included age, sex, self-reported race, and years of education. Health status-related covariates included cognitive functioning, functional status, depression, and body mass index, exercise (total number of occasions per week on which at least 15 minutes were performed for 8 activities), smoking (never, past, current), and alcohol use (never, past, current). Cognitive function was evaluated using the Cognitive Abilities Screening Instrument (CASI) [38], a 40-item test of global cognitive functioning, scaled such that the entire ACT cohort at baseline had a mean of 100 and standard deviation (SD) of 15. Self-reported functional status was measured using number of limitations in ADLs and instrumental activities of daily living (IADLs). Total number of ADL limitations was based on the participant's reported difficulty performing six ADLs (walking around inside the home, bathing/showering, dressing themselves, getting out of bed or a chair, feeding themselves, and using a toilet). Total number of IADL limitations was based on the participants reported difficulty in five IADLs (shopping, doing light housework, preparing meals, using a telephone, and managing money). Depressive symptoms were measured using the Center for Epidemiologic Studies Depression scale (CES-D) [39]. Scores were based on a standardized 10-question version [40]; each question contributed 0–3 points for possible range of 0 to 30 points. Height and weight were measured at each study visit. Body mass index was calculated from weight and height (kg/m^2^) and categorized as underweight (<18.5), normal (18.5 to 24.9), overweight (25–29.9), and obese (30+). Participants were asked whether a doctor had ever told them they had cancer, cardiovascular disease, cerebrovascular disease, diabetes, hypertension, or rheumatoid or osteoarthritis. We did not obtain information on number of medications.

### Statistical analysis

We used descriptive statistics to characterize the study population according to SRH. We described trends in PPF and SRH via their association with age. First, we estimated associations between baseline SRH and age-related change in PPF using linear mixed models with random intercepts to account for within-subject correlation. The primary exposures were age, baseline SRH, and their interactions. The outcome was the PPF score at each study visit. The interaction between age and baseline SRH allowed us to make inference on modifications to the relationship between age and PPF that were attributable to SRH. As sensitivity analyses, we re-ran models with missing PPF imputed first as the lowest PPF score and then as the highest PPF score. We also explored whether baseline SRH was associated with individual component measures of PPF.

Next, we estimated associations between baseline PPF and age-related change in SRH, repeating analyses but using a logistic link function in a generalized linear mixed model. Healthy SRH was the outcome and the primary exposures were age, quartiles of baseline PPF, and their interactions. Inference was based on the interaction between age and SRH, which described the extent to which the age-related differences in odds of healthy SRH were modified by baseline PPF. We also re-ran models with missing SRH values imputed as unhealthy SRH and then as healthy SRH.

We investigated three levels of time-varying covariate adjustment: Model 1 was adjusted for participant age at baseline; Model 2 was additionally adjusted for sex, race, education, cognitive functioning, limitations in ADLs and IADLS, depressive symptoms, body mass index, exercise, smoking status, and alcohol use; and Model 3 was additionally adjusted for chronic health conditions (cancer, cardiovascular disease, cerebrovascular disease, diabetes, hypertension, or arthritis). Potential confounders were selected a priori as factors previously found to be associated with SRH and PPF [21], [28]. Values of SRH adjusted for other health indicators may reflect subjective and contextual evaluations and response styles [1]. We pre-specified Model 2 as the primary analysis.

To describe longitudinal trajectories across follow-up we report the estimated mean outcome for each level of the primary exposure at age 75 (corresponding to the average age at baseline for the sample) and the estimated annual change in the outcome for each level of the primary exposure from each model. In Models 2 and 3, we estimated the mean at age 75 based on regression model estimates using indirect standardization to account for possible confounding by other covariates included in the model. We used graphical analyses to illustrate estimated age-related trends in SRH and PPF. Model fit was assessed with residual plots. Analyses were conducted using R (version 2.12.1). All tests were two-sided with α = 0.05.

## Results

As of July 25, 2011, data were available on 4,513 participants without dementia who had ever been enrolled in the ACT study, of which 4,411 participants were aged 65–89. Age-eligible participants were excluded if they were missing SRH, PPF, or covariate data at all visits. The final study sample included 3,610 ACT participants (81.8% of 4,411 age-eligible participants) followed on average for 4.8 (SD: 4.4) years. Excluded age-eligible participants were primarily missing PPF (n = 326) and/or health conditions (n = 497); they tended to have higher education, more limitations in ADLs and IADLs, more health conditions, and shorter follow-up than included participants (Table S1). At the baseline visit in this analysis (the initial visit for>90%), participants averaged 74.5 (SD: 5.8) years of age and 14.3 (SD: 3.1) years of education; approximately 60% were female and 90% were white. There were 1,517 participants (41.7%) who died during follow-up. At baseline, 13% of participants had excellent SRH, 33% had very good SRH, 39% had good SRH, 14% had fair SRH, and 2% had poor SRH. The average PPF score was 12.5 (SD: 2.5). On average, participants with unhealthy SRH were older, had worse cognition and more depressive symptoms, and reported functional limitations and comorbidities; and they were more likely to have died during follow-up (Table 2).

**Table 2:** Participant baseline characteristics by self-rated health (N = 3,610).

Participant Characteristics	Excellent/Very Good/Good SRH (n = 3,062)	Fair/Poor SRH (n = 548)
	Mean (SD)	Mean (SD)
Age	74.2 (5.8)	75.8 (5.9)
Cognitive Functioning		
CASI^a^	105.0 (10.5)	100.5 (10.3)
Depressive Symptoms		
CESD Score	3.4 (3.7)	6.6 (5.3)
Exercise		
Occasions per week of 15 minutes	5.6 (4.6)	4.1 (4.2)
	N (%)	N (%)
Female	1,796 (58.7)	319 (58.2)
Race		
White	2,819 (92.1)	467 (85.2)
Black	106 (3.5)	43 (7.8)
Asian	94 (3.1)	25 (4.6)
Other	43 (1.4)	13 (2.4)
Education		
<High school	295 (9.6)	109 (19.9)
Completed high school	689 (22.5)	177 (32.3)
At least some college	2078 (67.8)	262 (47.8)
ADL limitations (out of 6)		
0	2,582 (84.3)	318 (58.0)
1	382 (12.5)	125 (22.8)
≥2	98 (3.2)	105 (19.2)
IADL limitations (out of 5)		
0	2,770 (90.8)	374 (68.2)
1	234 (7.6)	96 (17.5)
≥2	49 (1.6)	78 (14.2)
Body Mass Index		
Underweight	27 (<1)	10 (1.8)
Normal	1,023 (33.4)	161 (29.4)
Overweight	1,264 (41.3)	210 (38.3)
Obese	748 (24.4)	167 (30.5)
Alcohol Use		
Never	565 (18.5)	145 (26.5)
Former	712 (23.3)	184 (33.6)
Current	1,785 (58.3)	219 (40.0)
Smoking		
Never	1,478 (48.2)	235 (42.9)
Former	1,425 (46.5)	270 (49.3)
Current	159 (5.2)	43 (7.8)
Health Conditions^b^		
None	723 (23.6)	54 (9.9)
One	1,141 (37.2)	137 (25.0)
Two or more	1,198 (39.1)	357 (65.1)
Death during follow-up	1,164 (38.0)	343 (62.6)

Abbreviations: SRH, self-rated health; CASI, Cognitive Abilities Screening Test; CESD, Center for Epidemiological Studies Depression Scale; ADL, Activities of Daily Living; IADL, Instrumental Activities of Daily Living.

a Scores are scaled such that at baseline the mean score for the entire ACT cohort was 100 and the standard deviation was 15.

b Health conditions included cancer, cerebrovascular disease, cardiovascular disease, diabetes, hypertension, and arthritis.

### Baseline SRH and Age-Related Changes in PPF

Among participants with at least one follow-up visit (n = 2,691), the average change in PPF score was −1.9 points over follow-up (SD: 2.7). Frequency and percentage of participants by trajectories of PPF are shown in Table 3. For a majority of participants (∼70%), PPF declined by at least one point over follow-up (Table 3). However, many participants had fluctuations in PPF over time. In initial descriptive analyses, the relationship between PPF and age was approximately linear, on average.

**Table 3:** Trajectories^a^ in PPF and SRH level over follow-up for participants with two or more visits (N = 2,691).

	No change	Declined	Improved	Fluctuated, overall decline	Fluctuated, other
**PPF score**	184 (6.8)	944 (35.1)	265 (9.8)	900 (33.4)	398 (14.8)
**SRH** b	2052 (76.3)	259 (9.6)	137 (5.1)	32 (<1)	211 (7.1)

a No change, declined, and improved categories represent trajectories with one pattern; fluctuated categories represent trajectories with both decline and improvement (with either overall decline or another pattern).

b dichotomized (healthy vs. unhealthy).

Note: N(% of 2,691) shown for each category.

**Figure 2:**
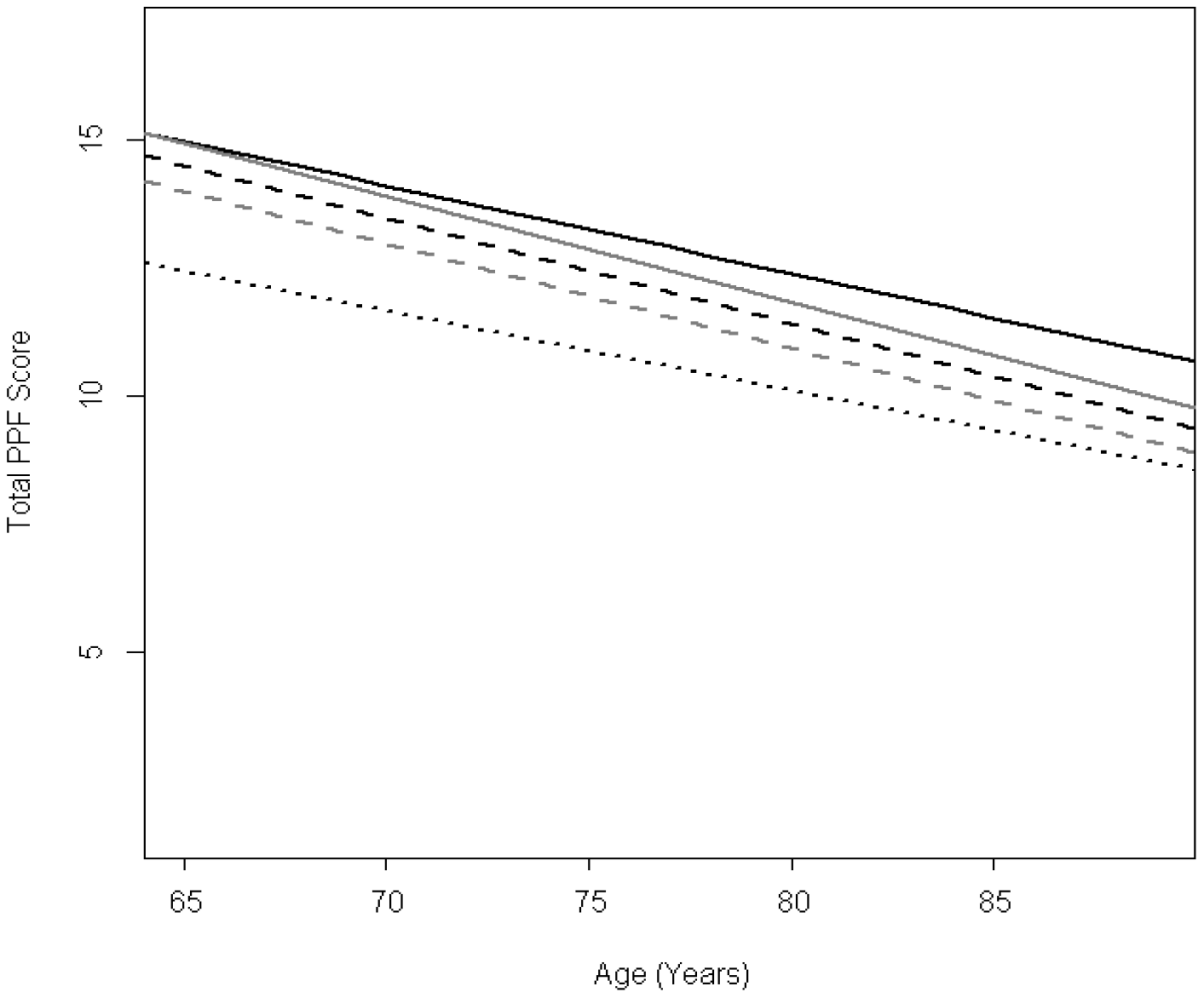
Population mean trajectories of performance-based physical functioning (PPF) score modified by self-rated health (SRH). Average trends in PPF for adults aged 65–89 were estimated from a linear mixed model adjusted for age at baseline, sex, race, education, cognitive functioning, depressive symptoms, functional limitations, body mass index, alcohol use, smoking status, and exercise. SRH levels are depicted as: excellent = black solid, very good = grey solid, good = black dashed, fair = grey dashed, poor = black dotted. PPF scores (y-axis) ranged from 0 to 16; higher scores corresponded to better performance.

In multivariable linear mixed models, poorer baseline SRH was associated with poorer mean PPF at age 75 (Table 4). On average, PPF declined with age even after adjustment for demographics and health status. There was a significant difference in rate of decline of PPF across levels of SRH (*P* = 0.02) (Table 4). Compared to participants with excellent baseline SRH, the average annual decline in PPF was slightly more rapid for participants with very good, good, and fair baseline SRH (Table 4, Figure 2). For participants with poor SRH, average annual decline was similar to participants with excellent SRH (Table 4). Results were similar across all models (Table S2). Very few participants with poor SRH at baseline (n = 21) had multiple visits; in examining PPF trajectories, we found that 9.5% had no change, 42.9% declined, 28.6% improved, and 19.0% fluctuated over follow-up. In exploratory analyses, annual change in odds of better function for individual PPF components was not consistently lower for poorer SRH (Table S3).

**Table 4:** Linear mixed model results for the associations between baseline SRH and age-related changes PPF.^a^

Mean PPF at age 75 by SRH^b^			
	Mean	95% CI	*P*-value^c^
Excellent	13.02	12.85, 13.19	<0.001
Very Good	12.63	12.53, 12.74	
Good	12.21	12.11, 12.31	
Fair	11.73	11.54, 11.91	
Poor	10.64	10.08, 11.21	

Annual rate of change in PPF by SRH level			
	*β*	95% CI	*P*-value^c^
Excellent	−0.17	−0.19, −0.15	0.02
Very Good	−0.21	−0.22, −0.19	
Good	−0.21	−0.22, −0.19	
Fair	−0.21	−0.23, −0.18	
Poor	−0.16	−0.24, −0.07	

Abbreviations: PPF, performance-based physical function; SRH, self-rated health.

a Adjusted for age at baseline, sex, race, education, cognitive functioning, depressive symptoms, functional limitations, body mass index, alcohol use, smoking status, and exercise.

b Estimates are standardized to the distribution of all covariates included in the model via indirect standardization.

c P-values are for omnibus Wald test of any difference across categories of SRH.

In sensitivity analyses, inference on variation in rates of decline in relation to baseline SRH was unchanged when all missing PPF values were imputed as the minimum, but when all missing PPF values were imputed as the maximum the mean rate of decline of PPF was slower for individuals with less than excellent baseline SRH (data not shown).

### Baseline PPF and Age-Related Changes in SRH

**Figure 3:**
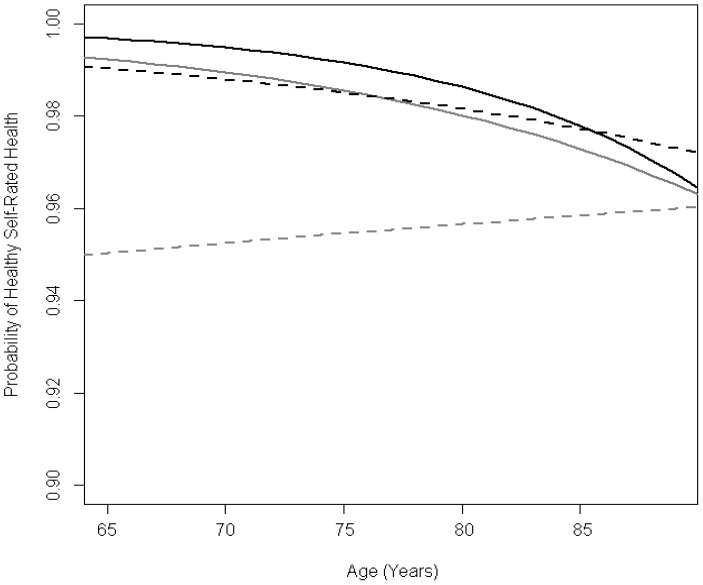
Population mean change in probability of excellent/very good/good (healthy) self-rated health (SRH) modified by quartiles of performance-based physical functioning (PPF). Average trends in PPF for adults aged 65–89 were estimated from a generalized linear mixed model adjusted for age at baseline, sex, race, education, cognitive functioning, depressive symptoms, functional limitations, body mass index, alcohol use, smoking status, and exercise. PPF Quartiles are depicted as: highest = black solid, upper-middle = grey solid, lower-middle = black dashed, lowest = grey dashed.

Among 2,691 participants with at least one follow-up visit the vast majority (>80%) remained stable in SRH (healthy vs unhealthy) during follow-up (Table 3). In multivariable generalized linear mixed models, lower baseline PPF quartiles were associated with lower probability of healthy SRH at age 75 (Table 5). There was a significant difference in change of odds of healthy SRH across PPF quartiles (*P*<0.001) (Table 5). For those in the highest quartile of baseline PPF there was a 9% decrease in odds of healthy SRH per year (OR = 0.91). Participants in the upper middle and lower middle quartiles experienced a slower decrease in odds of healthy SRH (4 and 6% decrease per year, respectively) (Table 5). In adjusted models, odds of healthy SRH increased with age, for those in the lowest quartile (1% increase per year). This resulted in convergence of the estimated probability of healthy SRH across levels of baseline PPF for older compared to younger ages (Figure 3). Results were similar across all models (Table S4). Sensitivity analyses produced similar results when missing SRH values were imputed as excellent or as poor (data not shown).

**Table 5:** Generalized linear mixed model results for the associations between baseline PPF and age-related changes in SRH.^a^

Probability of healthy SRH at age 75 by PPF quartile^b^			
	Prob	95% CI	*P*-value^c^
Highest	0.95	0.95, 0.96	<0.001
Upper Middle	0.93	0.92, 0.94	
Lower Middle	0.93	0.92, 0.94	
Lowest	0.84	0.81, 0.87	

Annual multiplicative rate of change in odds of healthy SRH by PPF quartile			
	OR	95% CI	*P*-value^c^
Highest	0.91	0.89, 0.93	<0.001
Upper Middle	0.94	0.91, 0.97	
Lower Middle	0.96	0.93, 0.99	
Lowest	1.01	0.97, 1.05	

Abbreviations: PPF, performance-based physical function; SRH, self-rated health.

a Adjusted for age at baseline, sex, race, education, cognitive functioning, depressive symptoms, functional limitations, body mass index, alcohol use, smoking status, and exercise.

b Estimates are standardized to the distribution of all covariates included in the model via indirect standardization.

c P-values are for omnibus Wald test of any difference across categories of SRH.

## Discussion

We investigated bi-directional longitudinal relationships between SRH and physical function among older adults without dementia, with adjustment for multiple health indicators. We found that lower baseline SRH was associated with lower PPF at age 75. Very good, good, or fair baseline SRH was associated with faster decline of PPF compared to excellent baseline SRH. Although we found that lower baseline PPF was associated with lower odds of healthy SRH at age 75, lower baseline PPF was associated with increasing odds of healthy SRH over time. This resulted in little difference in odds of healthy SRH at older ages.

Our results are consistent with previous studies that found fair/poor SRH to be associated with faster decline in timed gait [12], [13]. We extend these findings to a measure of overall physical function. Furthermore, we found associations with even intermediate levels of SRH, which has shown graded associations with risk of mortality and self-reported functional limitations [7], [9]. SRH may reflect a number of underlying constructs. SRH may represent an individual's superior knowledge of their own health over objective measures [2], [9]. Alternatively, SRH may reflect psychosocial resources and health behaviors that influence changes in function [41], [42]. Contrary to our expectations, poor baseline SRH did not predict more rapid decline in PPF compared to excellent SRH. This estimate may not be precise since very few participants with poor SRH had multiple visits. However, participants with poor SRH at baseline were more likely to have improved PPF scores in subsequent visits compared to those with higher levels of SRH. Perhaps for some participants poor SRH reflected acute or transient health effects that improved at later visits. On the other hand, participants with poor SRH at baseline were more likely to have died so there could also have been a bias toward follow-up of participants with poor SRH who had temporary health problems. Participants with poor SRH who had lasting impairments in functioning may have been more likely to die or be unable to return for additional study visits.

We provide new evidence describing the longitudinal change in healthy SRH according to baseline physical function. Our results complement prior findings that poor physical function did not predict decline in SRH [22], and that associations between functional status and SRH weakened with age [20]. Current level of function may be a stronger determinant of SRH than prior level of function [20]. Alternatively, these results may be due to regression to the mean, the statistical phenomenon where extreme measurements tend to be closer to the mean on a second measurement [43]; other studies have found such trends in SRH [44], [45]. Given that a large number of participants had fluctuating PPF trajectories, it is possible that non-uniform patterns of PPF over time explains the lack of predictive power of baseline PPF for subsequent changes in SRH. Another possible explanation is that with age, individuals may lower their expectations of good health after experiencing health declines [46]. This phenomenon, known as “response-shift”, could differ across levels of physical functioning. Participants with worse baseline physical function may have lowered expectations for their health over time resulting in stable or higher SRH at subsequent visits compared to those with better function. On the other hand, participants with high levels of physical functioning may have had higher expectations for their health, and age-related decline in health may be more discordant to their expectations leading to more rapid declines in SRH.

Although our results suggest that SRH and physical function are interconnected, independent of demographics and health outcomes, worse baseline physical function did not predict decline in SRH in a reciprocal fashion as worse baseline SRH predicted decline in physical function. People with less than excellent SRH may be at an increased risk for future disability [11] and mortality [7], [9] with decline in physical function as an intermediary [12], [13]. Since overall decline in physical function was common and was associated with lower SRH, future research should determine whether decline in physical function could be prevented among individuals with lower SRH. Multi-component exercise interventions may effectively prevent disability in frail adults [51], and could be used to target individuals by their SRH level. Conversely, after adjustment for current health status, changes in SRH with age may be due to a more dynamic process that reflects changing self-assessment. Older adults consider a variety of factors when assessing their health including functional status, health behaviors and psychosocial factors; however the importance of each factor may differ according to an individual's demographics, psychological outlook, physical health status, and question framing [17], [47], [48]. In future research, it may be important to investigate whether the relationships between SRH and PPF differ by subgroups and response styles.

Our study has several limitations. Generalizability may be limited because participants were predominantly white and well educated. Participants in poor health may have been more likely to die, drop-out earlier, or skip visits, which may have attenuated associations between SRH and PPF. Due to our interest in the subjective processes behind SRH rather than global health status, our measure of SRH did not incorporate death, unlike an alternative coding of SRH [24]. Some ACT participants were excluded from this analysis due to missing data, especially in our PPF measures, which may not be assessed on participants with disabilities, poor health, or impaired cognition. This may have biased our results towards participants with better physical function and longer follow-up. However, there is excellent follow-up of ACT participants (completeness of follow-up index estimated to be 95.6% [49]) and sensitivity analyses in which missing PPF was imputed as poor gave similar results to the primary analyses.

This study also has several important strengths, including a large, representative sample of community-dwelling older adults with extensive follow-up with multiple measures of health indicators. We used a longitudinal analysis, which allowed us to average over fluctuations in individual measurements to help capture long-term trends in PPF and SRH. Since many participants had fluctuating PPF measures, longitudinal modelling of repeated PPF measures can help uncover trends that would otherwise be obscured. We recommend this approach for future studies of PPF and SRH. Additionally, we investigated the associations between SRH and an objective measure of overall physical function eliminating potential confounding arising in subjective measures of function [50]. Our composite measure of PPF is simple to assess and clinically relevant since interventions to improve overall function may be more effective at preventing disability than single components [51]. However, there are many tests of physical function [18]; future research should investigate optimal measures. We selected an analysis strategy that is robust to data missingness that can be predicted based on prior observations [52]. Furthermore, we conducted sensitivity analyses, which generally supported our results except in the unlikely situation when PPF measurements were more likely to be missing for those with good function. We tested specific hypotheses regarding the relationships between SRH and PPF; however, future studies could explore how multiple factors influence change in SRH and PPF as well as their relative impact.

In conclusion, we found longitudinal associations between SRH and PPF in a large prospective cohort study of older adults. However, there appears to be a complex relationship between SRH and PPF. Future research should also assess the mechanisms through which SRH may affect physical function as well as disentangle factors that influence longitudinal change in SRH. As a strong predictor of mortality and functional decline, SRH provides a simple tool that can be used to identify individuals at higher risk of future poor health outcomes. It is unclear whether future poor health outcomes can be prevented among individuals with lower SRH. However, interventions to maintain physical function may be especially beneficial for those with less than excellent SRH.

## Supporting Information

Table S1 **Baseline Characteristics of Included and Excluded ACT Participants Aged 65–89 (N = 4,411).**
(DOC)

Table S2 **Linear Mixed Model Results for the Association between Baseline SRH and Longitudinal PPF.**
(DOC)

Table S3 **Generalized Linear Mixed Effects Model Results for the Association between Baseline SRH and Odds of Better Function^a^ for Individual Components of PPF.** Estimates of individual PPF components at age 75 are standardized to the distribution of all covariates included in the model via indirect standardization.
(DOC)

Table S4 **Generalized Linear Mixed Model Results for the Association between Baseline PPF Quartile and Odds of Excellent/Very Good/Good (“healthy”) SRH.**
(DOC)
