# Supplementary material for: Longitudinal Associations between Self-Rated Health and Performance-Based Physical Function in a Population-Based Cohort of Older Adults
Source: PLoS One. 2014 Nov 3;9(11):e111761. doi: 10.1371/journal.pone.0111761 (PMC4218810; doi:10.1371/journal.pone.0111761)
Supplement: Table S1 — Baseline Characteristics of Included and Excluded ACT Participants Aged 65–89 (N = 4,411). (DOC) [file pone.0111761.s001.doc]

| **Supporting Information Table S1**. Baseline Characteristics of Included and Excluded ACT Participants Aged 65-89 (N=4,411). | | | |
| --- | --- | --- | --- |
|  | **Included** |  | **Excluded** |
| **Participant Characteristics** | **(n= 3,610)** |  | **(n= 801)** |
|  | **Mean (SD)** |  | **Mean (SD) [n missing]** |
| Age | 74.5 (5.8) |  | 74.2 (6.6) |
| Cognitive Functioning  CASI^a^ | 104.3 (10.6) |  | 104.3 (10.8) [17] |
| Depressive Symptoms  CESD Score | 3.9 (4.2) |  | 3.5 (4.6) [67] |
| Exercise  Occasions per week of 15 minutes | 5.4 (4.5) |  | 4.9 (4.7) [11] |
| Follow-up time  Years | 4.8 (4.4) |  | 1.5 (2.0) |
|  | **N (%)** |  | **N (%) [n missing]** |
| Female | 2,115 (58.6) |  | 568 (58.2) |
| Race  White  Black  Asian  Other | 3,286 (91.0)  149 (4.1)  119 (3.3)  56 (1.6) |  | [8]  715 (90.2)  30 (3.8)  39 (4.9)  9 (1.1) |
| Education  < High school  Completed high school  At least some college | 404 (11.2)  866 (24.0)  2340 (64.8) |  | [1]  49 (6.1)  115 (14.4)  636 (79.5) |
| ADL limitations (out of 6)  0  1  ≥2 | 2,900 (80.3)  507 (14.0)  203 (5.6) |  | [19]  551 (70.5)  104 (13.3)  127 (16.2) |
| IADL limitations (out of 5)  0  1  ≥2 | 3,153 (87.3)  330 (9.1)  127 (3.5) |  | [17]  588 (68.2)  96 (17.5)  100 (14.2) |
| Body Mass Index |  |  | [83] |
| Underweight  Normal  Overweight  Obese | 37 (1.0)  1,184 (32.8)  1,474 (40.8)  915 (25.3) |  | 10 (1.4)  232 (32.3)  271 (37.7)  205 (28.6) |
| Alcohol Use |  |  | [10] |
| Never  Former  Current | 710 (19.7)  896 (24.8)  2,004 (55.5) |  | 158 (20.0)  189 (23.9)  444 (56.1) |
| Smoking |  |  | [10] |
| Never  Former  Current | 1,713(47.4)  1,695 (47.0)  202 (5.6) |  | 373 (47.2)  384 (48.5)  34 (4.3) |
| Health Conditions^b^ |  |  | [497] |
| None | 777 (21.5) |  | 19 (9.9) |
| One | 1278 (35.4) |  | 78 (25.0) |
| Two or more | 1,555 (43.1) |  | 207 (65.1) |
| Abbreviations: SRH, self-rated health; CASI, Cognitive Abilities Screening Test; CESD, Center for Epidemiological Studies Depression Scale; ADL, Activities of Daily Living; IADL, Instrumental Activities of Daily Living.  ^a^Scores are scaled such that at baseline the mean score for the entire ACT cohort was 100 and the standard deviation was 15.  ^b^Health conditions included cancer, cerebrovascular disease, cardiovascular disease, diabetes, hypertension, and arthritis. | | | |
