# Supplementary material for: Longitudinal Associations between Self-Rated Health and Performance-Based Physical Function in a Population-Based Cohort of Older Adults
Source: PLoS One. 2014 Nov 3;9(11):e111761. doi: 10.1371/journal.pone.0111761 (PMC4218810; doi:10.1371/journal.pone.0111761)
Supplement: Table S2 — Linear Mixed Model Results for the Association between Baseline SRH and Longitudinal PPF. (DOC) [file pone.0111761.s002.doc]

**Supporting Information Table S2.** Linear Mixed Model Results for the Association between Baseline SRH and Longitudinal PPF.

|  | **Model 1^a^** | | |  | **Model 2^b^** | | |  | **Model 3^c^** | | |
| --- | --- | --- | --- | --- | --- | --- | --- | --- | --- | --- | --- |
| **Mean PPF at age 75 by SRH^d^** | | | | | | |  |  |  |  |  |
|  | **PPF** | **95% CI** | ***P* value^e^** |  | **PPF** | **95% CI** | ***P* value^e^** |  | **PPF** | **95% CI** | ***P* value^e^** |
| Excellent | 13.40 | 13.21, 13.59 | <0.001 |  | 13.02 | 12.85, 13.19 | <0.001 |  | 12.88 | 12.71, 13.05 | <0.001 |
| Very Good | 12.82 | 12.7, 12.94 |  |  | 12.63 | 12.53, 12.74 |  |  | 12.56 | 12.46, 12.67 |  |
| Good | 12.16 | 12.05, 12.28 |  |  | 12.21 | 12.11, 12.31 |  |  | 12.21 | 12.1, 12.31 |  |
| Fair | 11.07 | 10.87, 11.28 |  |  | 11.73 | 11.54, 11.91 |  |  | 11.82 | 11.64, 12.01 |  |
| Poor | 9.14 | 8.51, 9.76 |  |  | 10.64 | 10.08, 11.21 |  |  | 10.77 | 10.21, 11.33 |  |
| **Annual rate of change in PPF by SRH level** | | | | | | |  |  |  |  |  |
|  | ***β*** | **95% CI** | ***P* value^e^** |  | ***β*** | **95% CI** | ***P* value^e^** |  | ***β*** | **95% CI** | ***P* value^e^** |
| Excellent | -0.20 | -0.22, -0.18 | <0.001 |  | -0.17 | -0.19, -0.15 | 0.02 |  | -0.15 | -0.17, -0.13 | 0.03 |
| Very Good | -0.24 | -0.26, -0.23 |  |  | -0.21 | -0.22, -0.19 |  |  | -0.18 | -0.20, -0.17 |  |
| Good | -0.25 | -0.27, -0.24 |  |  | -0.21 | -0.22, -0.19 |  |  | -0.18 | -0.20, -0.17 |  |
| Fair | -0.25 | -0.28, -0.23 |  |  | -0.21 | -0.23, -0.18 |  |  | -0.18 | -0.21, -0.16 |  |
| Poor | -0.22 | -0.31, -0.13 |  |  | -0.16 | -0.24, -0.07 |  |  | -0.14 | -0.22, -0.06 |  |

Abbreviations: PPF, performance-based physical functioning; SRH, self-rated health.

**^a^**Adjusted for age at baseline.

**^b^**Model 1 plus sex, race, education, cognitive functioning, depressive symptoms, functional limitations, body mass index, alcohol use, smoking status, and exercise.

**^c^**Model 2 plus cancer, cerebrovascular disease, cardiovascular disease, diabetes, hypertension, and arthritis.

**^d^**Estimates are standardized to the distribution of all covariates included in the model via indirect standardization.

**^e^**P-values are for omnibus Wald test of any difference across categories of SRH.
