# Supplementary material for: Longitudinal Associations between Self-Rated Health and Performance-Based Physical Function in a Population-Based Cohort of Older Adults
Source: PLoS One. 2014 Nov 3;9(11):e111761. doi: 10.1371/journal.pone.0111761 (PMC4218810; doi:10.1371/journal.pone.0111761)
Supplement: Table S3 — Generalized Linear Mixed Effects Model Results for the Association between Baseline SRH and Odds of Better Functiona for Individual Components of PPF. Estimates of individual PPF components at age 75 are standardized to the distribution of all covariates included in the model via indirect standardization. (DOC) [file pone.0111761.s003.doc]

| **Supporting Information Table S3.** Generalized Linear Mixed Effects Model Results for the Association between Baseline SRH and Odds of Better Function^a^ for Individual Components of PPF. Estimates of individual PPF components at age 75 are standardized to the distribution of all covariates included in the model via indirect standardization. | | | | | | | | | | | | | | | | |
| --- | --- | --- | --- | --- | --- | --- | --- | --- | --- | --- | --- | --- | --- | --- | --- | --- |
|  | **Walking Speed**^b^ | | | | **Chair Rises**^b^ | | | | **Standing Balance**^b^ | | | | **Grip Strength**^b^ | | | |
| **Probability of better function at age 75 by SRH** | | | | | | | | | | | | | | | | |
|  | **Prob** | **95% CI** | ***P*-value**^c^ | | **Prob** | **95% CI** | ***P-*value**^c^ | | **Prob** | **95% CI** | ***P-*value**^c^ | | **Prob** | **95% CI** | ***P-*value**^c^ | |
| Excellent | 0.95 | 0.94, 0.97 | | <0.001 | 0.87 | 0.85, 0.89 | | <0.001 | 0.86 | 0.84, 0.89 | | <0.001 | 0.85 | 0.82, 0.89 | | <0.001 |
| Very Good | 0.93 | 0.92, 0.94 | |  | 0.82 | 0.81, 0.84 | |  | 0.85 | 0.84, 0.87 | |  | 0.79 | 0.76, 0.82 | |  |
| Good | 0.88 | 0.87, 0.90 | |  | 0.77 | 0.75, 0.79 | |  | 0.81 | 0.79, 0.82 | |  | 0.71 | 0.68, 0.74 | |  |
| Fair | 0.82 | 0.78, 0.85 | |  | 0.72 | 0.68, 0.75 | |  | 0.77 | 0.74, 0.81 | |  | 0.65 | 0.59, 0.71 | |  |
| Poor | 0.69 | 0.56, 0.83 | |  | 0.62 | 0.47, 0.77 | |  | 0.72 | 0.61, 0.83 | |  | 0.65 | 0.48, 0.82 | |  |
| **Annual multiplicative rate of change**  **in odds of healthy SRH by PPF quartile** | | | | | | | | | | | | | | | | |
|  | **OR** | **95% CI** | ***P*-value**^c^ | | **OR** | **95% CI** | ***P-*value**^c^ | | **OR** | **95% CI** | ***P-*value**^c^ | | **OR** | **95% CI** | ***P-*value**^c^ | |
| Excellent | 0.82 | 0.79, 0.85 | | <0.001 | 0.93 | 0.90, 0.96 | | 0.55 | 0.87 | 0.84, 0.89 | | 0.02 | 0.75 | 0.73, 0.78 | | 0.003 |
| Very Good | 0.80 | 0.78, 0.81 | |  | 0.92 | 0.90, 0.94 | |  | 0.88 | 0.86, 0.89 | |  | 0.72 | 0.71, 0.74 | |  |
| Good | 0.85 | 0.83, 0.86 | |  | 0.94 | 0.92, 0.95 | |  | 0.89 | 0.88, 0.91 | |  | 0.76 | 0.74, 0.77 | |  |
| Fair | 0.84 | 0.82,0.87 | |  | 0.95 | 0.92,0.98 | |  | 0.92 | 0.89, 0.95 | |  | 0.78 | 0.75, 0.81 | |  |
| Poor | 1.04 | 0.93,1.16 | |  | 0.94 | 0.84,1.04 | |  | 0.90 | 0.82, 0.98 | |  | 0.81 | 0.72, 0.92 | |  |
| Abbreviations: OR, odds ratio; PPF, performance-based physical functioning; SRH, self-rated health.  ^a^Score of 3-4 for walking speed, chair rises, and grip strength; score of 4 for balance (due to distribution)  ^b^Adjusted for age at baseline, sex, race, education, cognitive functioning, depressive symptoms, functional limitations, body mass index, alcohol use, smoking status, and exercise.  ^c^*P*-values are for omnibus Wald test of any difference across categories of SRH. | | | | | | | | | | | | | | | | |
