# Supplementary material for: Longitudinal Associations between Self-Rated Health and Performance-Based Physical Function in a Population-Based Cohort of Older Adults
Source: PLoS One. 2014 Nov 3;9(11):e111761. doi: 10.1371/journal.pone.0111761 (PMC4218810; doi:10.1371/journal.pone.0111761)
Supplement: Table S4 — Generalized Linear Mixed Model Results for the Association between Baseline PPF Quartile and Odds of Excellent/Very Good/Good (“healthy”) SRH. (DOC) [file pone.0111761.s004.doc]

**Supplementary Table S4**. Generalized Linear Mixed Model Results for the Association between Baseline PPF Quartile and Odds of Excellent/Very Good/Good ("healthy") SRH.

|  | **Model 1^a^** | | | |  | **Model 2^b^** | | | |  | **Model 3^c^** | | | |
| --- | --- | --- | --- | --- | --- | --- | --- | --- | --- | --- | --- | --- | --- | --- |
| **Probability of healthy SRH at age 75 by PPF quartile^d^** | | | | | | | | |  |  |  |  | |  |
|  | **Prob** | **95% CI** | ***P* value^e^** | |  | **Prob** | **95% CI** | ***P* value^e^** | |  | **Prob** | **95% CI** | ***P* value^e^** | |
| Highest | 0.98 | 0.97, 0.98 | | <0.001 |  | 0.95 | 0.95, 0.96 | | <0.001 |  | 0.94 | 0.93, 0.95 | | <0.001 |
| Upper Middle | 0.95 | 0.94, 0.96 | |  |  | 0.93 | 0.92, 0.94 | |  |  | 0.92 | 0.90, 0.93 | |  |
| Lower Middle | 0.94 | 0.93, 0.96 | |  |  | 0.93 | 0.92, 0.94 | |  |  | 0.92 | 0.90, 0.93 | |  |
| Lowest | 0.74 | 0.69, 0.80 | |  |  | 0.84 | 0.81, 0.87 | |  |  | 0.83 | 0.81, 0.86 | |  |
| **Annual multiplicative rate of change**  **in odds of healthy SRH by PPF quartile** | | | | | | | | | |  |  |  | |  |
|  | **OR** | **95% CI** | ***P* value^e^** | |  | **OR** | **95% CI** | ***P* value^e^** | |  | **OR** | **95% CI** | ***P* value^e^** | |
| Highest | 0.87 | 0.85, 0.88 | | <0.001 |  | 0.91 | 0.89, 0.93 | | <0.001 |  | 0.95 | 0.93, 0.97 | | <0.001 |
| Upper Middle | 0.90 | 0.88, 0.93 | |  |  | 0.94 | 0.91, 0.97 | |  |  | 0.98 | 0.95, 1.01 | |  |
| Lower Middle | 0.91 | 0.88, 0.93 | |  |  | 0.96 | 0.93, 0.99 | |  |  | 1.00 | 0.97, 1.03 | |  |
| Lowest | 0.96 | 0.92, 0.99 | |  |  | 1.01 | 0.97, 1.05 | |  |  | 1.05 | 1.01, 1.09 | |  |

Abbreviations: OR, odds ratio; PPF, performance-based physical functioning; SRH, self-rated health.

^a^Adjusted for age at baseline.

^b^Model 1 plus sex, race, education, cognitive functioning, depressive symptoms, functional limitations, body mass index, alcohol use, smoking status, and exercise.

^c^Model 2 plus cancer, cerebrovascular disease, cardiovascular disease, diabetes, hypertension, and arthritis.

^d^Estimates are standardized to the distribution of all covariates included in the model via indirect standardization.

^e^P-values are for omnibus Wald test of any difference across categories of SRH.
